# Periphilin self-association underpins epigenetic silencing by the HUSH complex

**DOI:** 10.1093/nar/gkaa785

**Published:** 2020-09-25

**Authors:** Daniil M Prigozhin, Christopher H Douse, Laura E Farleigh, Anna Albecka, Iva A Tchasovnikarova, Richard T Timms, Shun-ichiro Oda, Frank Adolf, Stefan M V Freund, Sarah Maslen, Paul J Lehner, Yorgo Modis

**Affiliations:** Molecular Immunity Unit, Department of Medicine, University of Cambridge, MRC Laboratory of Molecular Biology, Cambridge CB2 0QH, UK; Molecular Immunity Unit, Department of Medicine, University of Cambridge, MRC Laboratory of Molecular Biology, Cambridge CB2 0QH, UK; Molecular Immunity Unit, Department of Medicine, University of Cambridge, MRC Laboratory of Molecular Biology, Cambridge CB2 0QH, UK; Cambridge Institute of Therapeutic Immunology & Infectious Disease (CITIID), Department of Medicine, University of Cambridge, Cambridge CB2 0AW, UK; Molecular Immunity Unit, Department of Medicine, University of Cambridge, MRC Laboratory of Molecular Biology, Cambridge CB2 0QH, UK; Cambridge Institute of Therapeutic Immunology & Infectious Disease (CITIID), Department of Medicine, University of Cambridge, Cambridge CB2 0AW, UK; Cambridge Institute of Therapeutic Immunology & Infectious Disease (CITIID), Department of Medicine, University of Cambridge, Cambridge CB2 0AW, UK; Molecular Immunity Unit, Department of Medicine, University of Cambridge, MRC Laboratory of Molecular Biology, Cambridge CB2 0QH, UK; Cambridge Institute of Therapeutic Immunology & Infectious Disease (CITIID), Department of Medicine, University of Cambridge, Cambridge CB2 0AW, UK; Molecular Immunity Unit, Department of Medicine, University of Cambridge, MRC Laboratory of Molecular Biology, Cambridge CB2 0QH, UK; Cambridge Institute of Therapeutic Immunology & Infectious Disease (CITIID), Department of Medicine, University of Cambridge, Cambridge CB2 0AW, UK; NMR Facility, MRC Laboratory of Molecular Biology, Cambridge CB2 0QH, UK; Biological Mass Spectrometry & Proteomics Laboratory, MRC Laboratory of Molecular Biology, Cambridge CB2 0QH, UK; Cambridge Institute of Therapeutic Immunology & Infectious Disease (CITIID), Department of Medicine, University of Cambridge, Cambridge CB2 0AW, UK; Molecular Immunity Unit, Department of Medicine, University of Cambridge, MRC Laboratory of Molecular Biology, Cambridge CB2 0QH, UK; Cambridge Institute of Therapeutic Immunology & Infectious Disease (CITIID), Department of Medicine, University of Cambridge, Cambridge CB2 0AW, UK

## Abstract

Transcription of integrated DNA from viruses or transposable elements is tightly regulated to prevent pathogenesis. The Human Silencing Hub (HUSH), composed of Periphilin, TASOR and MPP8, silences transcriptionally active viral and endogenous transgenes. HUSH recruits effectors that alter the epigenetic landscape and chromatin structure, but how HUSH recognizes target loci and represses their expression remains unclear. We identify the physicochemical properties of Periphilin necessary for HUSH assembly and silencing. A disordered N-terminal domain (NTD) and structured C-terminal domain are essential for silencing. A crystal structure of the Periphilin-TASOR minimal core complex shows Periphilin forms an α-helical homodimer, bound by a single TASOR molecule. The NTD forms insoluble aggregates through an arginine/tyrosine-rich sequence reminiscent of low-complexity regions from self-associating RNA-binding proteins. Residues required for TASOR binding and aggregation were required for HUSH-dependent silencing and genome-wide deposition of repressive mark H3K9me3. The NTD was functionally complemented by low-complexity regions from certain RNA-binding proteins and proteins that form condensates or fibrils. Our work suggests the associative properties of Periphilin promote HUSH aggregation at target loci.

## INTRODUCTION

More than half of the human genome consists of transposable elements (TEs). TEs have evolved to fulfill important cellular functions. TEs drive the evolution of transcriptional networks by spreading transcription factor binding sites, promoters and other regulatory elements (1,2). TE-derived regulatory elements are particularly important in embryogenesis, when global hypomethylation promotes transcription. Key pluripotency-associated transcription factors involved in cell fate determination bind to sites within TEs (1). TE genes also serve as a genetic reservoir that can be coopted by the host. For example, TE-derived proteins catalyze V(D)J recombination (3) and syncytiotrophoblast fusion in placental development (1,4).

A subset of TEs can autonomously replicate through an RNA intermediate and reintegrate into the genome like retroviruses. Some of these TEs are endogenous retrovirus (ERV) genomes inherited from ancestral infections of the germline. The other type of autonomously replicating TE in humans are the non-viral LINE-1 (long interspersed nuclear element-1) retroelements. Active ERVs and LINE-1s are transcribed and encode reverse transcriptase and integrase enzymes, which convert the transcripts into DNA and reintegrate it into the host genome (1). This amplifying retrotransposition mechanism has allowed ERVs and L1s to accumulate in the human genome. Approximately 100 human LINEs are replication-competent and cause new integration events in 2–5% of the population (5,6).

Transcription and retrotransposition of TEs must be tightly regulated to prevent harmful gene expression and genome damage. Accumulation of TE transcripts is associated with autoimmune diseases including geographic atrophy, lupus and Sjögren's syndrome (5,7). Aberrant expression of proteins from the human ERV HERV-K is associated with cancer and neurodegeneration (8). Reactivation of ERVs and LINE-1s in somatic cells is also associated with cancer, through disruption of tumor suppressor genes or enhanced transcription of oncogenes (9,10). Disruption of protein coding sequences by transposition events is additionally linked to genetic disorders such as hemophilia and cystic fibrosis (9,10).

A central mechanism cells have evolved to control potentially pathogenic expression and transposition of TEs and infectious viruses alike is epigenetic silencing. Among the most important sources of epigenetic silencing in humans is the Human Silencing Hub (HUSH) complex, consisting of three proteins: Periphilin, TASOR and MPP8 (11). HUSH silences the genomes from newly integrated lentiviruses (11) as well as unintegrated retroviruses via the DNA-binding protein NP220 (12). Vpr and Vpx proteins from lentiviruses including HIV target HUSH for proteasomal degradation, demonstrating the importance of HUSH-dependent silencing in controlling lentiviral infection (13–15). HUSH also silences hundreds of transcriptionally-active or recently-integrated genomic sequences, with a degree of selectivity for full-length LINE-1s located in euchromatic environments, often within introns of actively transcribed genes (16,17). In the current model of HUSH-dependent repression HUSH spreads histone H3 lysine 9 trimethylation (H3K9me3), a transcriptionally repressive mark, by recruiting the H3K9 methyltransferase SETDB1 and its stabilizing factor ATF7IP to existing H3K9me3 marks via MPP8, which binds both H3K9me3 and ATF7IP (11,18). This read-write mechanism for H3K9me3 spreading by MPP8 and SETDB1 alone is insufficient for repression, however, as TASOR, Periphilin and portions of MPP8 each have functions other than binding H3K9me3 that are essential (11). HUSH silencing also requires MORC2, a DNA-binding ATPase thought to be a chromatin remodeler (19,20). The specific contributions of the three HUSH subunits in recognizing target loci and repressing their expression therefore remain unclear.

The biochemical and structural properties of the three HUSH subunits remain mostly unknown. In this study, we delineate the key structural and physicochemical attributes of Periphilin and how they contribute to HUSH function. Periphilin was originally identified as a highly insoluble nuclear protein cleaved by caspase-5 (21). N-terminal sequences contain the determinants for insolubility and the C-terminal region contains predicted α-helical heptad repeats proposed to form dimers based on a yeast two-hybrid assay (21). Periphilin is indispensable for development. In mice, homozygous deficiency of Periphilin is lethal early in embryogenesis and heterozygous deficiency is compensated by increased expression from the wild-type allele (22). Overexpression of Periphilin transcriptionally represses certain proteins causing cell cycle arrest (23,24). Isoform 2 of Periphilin, one of at least 8 isoforms, was identified in a gene-trap mutagenesis screen as a component of the HUSH complex that binds TASOR but not MPP8 (11,17). Curiously, some of the isoform diversity is driven by TE insertion into Periphilin coding sequences (25). Periphilin was also identified as an mRNA-binding protein in a screen of the protein-mRNA interactome in proliferating human cells (26). Here, we report the crystal structure of a Periphilin-TASOR minimal core complex and identify the key physicochemical properties of Periphilin necessary for HUSH complex assembly and epigenetic silencing. The Periphilin C-terminal region directs HUSH complex assembly by dimerizing and binding a single TASOR molecule through α-helical coiled-coil interactions. A disordered N-terminal domain (NTD) mediates self-aggregation through a sequence enriched in arginine and tyrosine residues. The sequence of the NTD is reminiscent of—and functionally complemented by—low-complexity regions from RNA-binding proteins, and from certain proteins that self-associate to form biomolecular condensates or phase separations. Our findings suggest Periphilin may contribute to the recognition and co- or post-transcriptional repression of HUSH target loci by binding and sequestering nascent transcripts. This work provides a foundation to design strategies to control HUSH activity, with important potential therapeutic applications.

## MATERIALS AND METHODS

### Protein sequence analysis

We used CIDER software developed for the analysis of intrinsically disordered proteins (27) to extract Periphilin sequence parameters including predicted structural disorder, charge and hydropathy.

### Protein expression vectors

Synthetic genes encoding codon-optimized Periphilin-1 isoform 2 (UniProt Q8NEY8-2, residues 285–374) and TASOR isoform 1 (UniProt Q9UK61-1, residues 1014–1095) were cloned sequentially into pRSF-Duet vector (Novagen) using HiFi Assembly (NEB), producing an N-terminally hexahistidine (His_6_)-tagged Periphilin and untagged TASOR fragments. These constructs were used to produce Periphilin-TASOR complex for biophysical characterization by NMR and native mass spectrometry (see below). Following identification of disordered regions by NMR, a shorter Periphilin variant (residues 292–367) was cloned, preceded by a His_6_ tag and a TEV protease cleavage site (ENLYFQG). This construct was used for crystallization and SEC-MALS of the Periphilin-TASOR complex. Full-length, 1–127, and 128–374 Periphilin codon-optimized for *Escherichia coli* were cloned into pET15b plasmid (Novagen), with N-terminal His_6_ tags followed by a thrombin protease cleavage site (LVPRGS); these constructs were used for biophysical characterization of the Periphilin N-terminus.

### Lentivirus complementation assay vectors

Wild-type Periphilin was cloned into lentiviral vector pHRSIN-PSFFV-V5-Periphilin-PPGK-Hygro as described (11). For Periphilin mutants L356R, L326A and L333A/I337A, the vector was digested with MluI and NotI to remove the residues 295–374 of the insert. Synthetic dsDNA fragments carrying the mutations were inserted into the gel-purified vector by HiFi Assembly. For the Δ1–127, Δ1–70, and Δ71–127 deletion mutants, the vector was digested with KpnI and NotI to remove the Periphilin sequence and one or two PCR fragments corresponding to the retained sequences were inserted by assembly. The NTD(DE>NQ), NTD(R>K) and NTD(Y>S) variants were generated by assembling a synthetic dsDNA encoding Periphilin residues 1–127 with the desired mutations with a PCR product encoding Periphilin residues 128–374 and KpnI/NotI-digested vector. Constructs with complementing sequences from other proteins were generated in the same manner but using a synthetic dsDNA encoding: FUS PLD (UniProt P35637, residues 2–214); SUP35 PrD (UniProt P05453, residues 5–135); ALYREF2 residues 17–67 (UniProt Q9JJW6.1), YBX3 residues 151–268 (UniProt P16989-1), and FUS RBD (UniProt P35637, residues 454–526).

### Cell lines and lentivirus production

HeLa cells carrying integrated GFP reporter—with or without Periphilin KO (11)—and HEK 293T cells were maintained in RPMI supplemented with 10% fetal calf serum, 50 U/ml penicillin, 50 μg/ml streptomycin. Lentiviruses were produced by cotransfection of HEK 293T cells at 95% confluence in six-well plates with 3 μg of the following plasmids at a 1:2:2 molar ratio: pMD2.G carrying glycoprotein VSV-G, pCMVΔ8.91 carrying replicative genes, and the pHRSIN-based lentiviral backbone containing hygromycin resistance and Periphilin. The plasmids were mixed with 200 μl serum-free medium, and 15 μl PEI, incubated at room temperature for 20 min and applied to cells. Media was exchanged 4 h post-transfection and supernatants containing lentiviruses were harvested 48 h post-transfection by filtering through a 0.45 μm filter and stored at −80°C.

### Reporter silencing assay

Reporter silencing activity of WT and mutant Periphilin was measured by infecting the Periphilin KO HeLa reporter cell line with lentiviruses carrying Periphilin variants and monitoring GFP fluorescence for 21 days post-transduction (11). Periphilin KO HeLa cells in 24-well plates were overlaid with 150 μl of lentiviral supernatants and 8 μg/ml polybrene and centrifuged for 90 min at room temperature at 1000 g. After 24 h incubation cells were trypsinized and seeded into flasks with selection media containing 400 μg/ml hygromycin. Fresh media was added every other day. Hygromycin was removed from the media after 7 days in culture. For flow cytometry cells were trypsinized, washed in PBS, counted, and resuspended at 1 × 10^6^ cells per ml in PBS supplemented with 2% fetal calf serum. GFP fluorescence was recorded with an Eclipse flow cytometer (iCYT) from >1 × 10^5^ cells per sample. The cells were gated on live single-cell population based on forward and side scatter in FlowJo (BD Life Sciences). The geometric mean of the GFP fluorescence of the whole live population was determined without further gating and values exported to Excel (see Supplementary Data Set S1). Since gene expression data are log-normal, we converted GFP fluorescence to percent repression with the formula: %GFP Repression = log_10_(GeoMeanPopulation)*m + *a*, where *m* = 100%/[log_10_(GeoMeanWT) – log_10_(GeoMeanKO)] and *b* = –m*log_10_(GeoMeanKO). This transformation assigned the Periphilin KO population a value of 0% repression and WT HeLa reporter cells (11) 100% repression. The values of *m* and *b* used were –59.7 and 196.1, respectively, for all experiments except those in Figure 6C, where they were –59.5 and 210.0. The difference in vertical offset was due to a laser upgrade on the instrument.

### Co-immunoprecipitation and Western immunoblotting

For co-immunoprecipitation (co-IP), cells were lysed in 1% NP-40 or IGPAL C-630 in TBS plus 10 mM iodoacetamide and protease inhibitors: 0.5 mM phenylmethylsulfonyl fluoride (PMSF) and benzonase (Sigma-Aldrich) or Complete Protease Inhibitor Cocktail (Roche), for 30 min. The cell lysate was centrifuged at 14 000 g for 10 min and the supernatant (Input) mixed with Protein A and IgG-sepharose resin along with primary antibody. The suspension was incubated for 2 h at 4°C and the resin was washed three times in lysis buffer. For western blotting, cells were lysed with lysis buffer containing 1% SDS for 30 min at room temperature. For SDS-PAGE analysis, Input and resins from co-IP or cell lysates for westerns were heated to 70°C in SDS sample buffer for 10 min and run on a polyacrylamide gel. Gels were blotted onto PVDF membranes (Millipore). Blots were blocked in 5% milk in PBS, 0.2% Tween-20 and incubated overnight with primary antibody diluted 1:5000 in blocking solution. As the Periphilin antibody was unable to detect its epitope under NP-40 lysis conditions, we used a mouse antibody against the V5 tag (Abcam, ab27671) as the primary antibody for Periphilin. For TASOR, the primary antibody was rabbit α-TASOR (Atlas, HPA006735). The primary antibody for loading controls was rabbit anti-actin (Abcam, ab219733). Blots were imaged with West Pico or West Dura (Thermo Fisher Scientific), or with the near-infrared system of a LI-COR Odyssey fluorescent scanner after incubation with DyLight 680- or 800-conjugated secondary antibodies (Thermo Fisher Scientific) at 1:10 000 dilution for 30 min at room temperature.

### Protein expression and purification

*Escherichia coli* BL21 (DE3) cells (New England BioLabs) were transformed with pRSF-DUET constructs expressing TASOR-Periphilin complex and selected on kanamycin plates. For native protein expression, overnight cultures were diluted 1:200 into 2 l of LB. Cultures were induced with 100 μM IPTG at OD_600_ 0.6, incubated at 37°C for 2 h, harvested, resuspended in 50 ml Buffer A (20 mM HEPES pH 7.4, 0.5 M NaCl, 0.5 mM TCEP) and frozen in liquid nitrogen. The cells were thawed at room temperature, supplemented with 1 μl benzonase (Sigma) and lysed by sonication. The lysates were clarified by centrifugation and filtering over a 0.45 μm filter. The clarified lysates were applied to 1-ml HisTrap columns (GE Healthcare), using one column per liter culture, washed with 20 column volumes of Buffer A and eluted in with 0.5 M imidazole in Buffer A. To purify non-cleavable His_6_-tagged Periphilin residues 285–374, the eluate was desalted into Buffer QA (20 mM NaCl, 20 mM HEPES pH 7.4, 0.5 mM TCEP) using a HiPrep desalting column (GE Healthcare), bound to MonoQ ion-exchange column (GE Healthcare) and eluted in a gradient of Buffer QA and BufferQB (1 M NaCl, 20 mM HEPES pH 7.4, 0.5 mM TCEP). Size-exclusion chromatography (SEC) on a Superdex 200 10/300 column (GE Healthcare) in PBS supplemented with 1mM TCEP and 0.05% sodium azide completed purification. For the ^15^N and ^15^N/^13^C-labeled protein expression, the overnight starter culture was grown in complete unlabeled minimal medium (M9) and used to inoculate (1:100 v/v) 800 ml complete labeled M9 media. Labeled proteins were purified as described above. For the construct expressing the TEV-cleavable His_6_-tagged Periphilin residues 292–367, Ni-affinity purification was followed by overnight digestion with TEV protease at 22°C, desalting into Buffer SA (20 mM NaCl, 20 mM Acetate pH 4.55, 0.5 mM TCEP), binding to a MonoS column, and elution against Buffer SB (1 M NaCl, 20 mM sodium acetate pH 4.55, 0.5 mM TCEP). SEC on a Superdex 200 column in 20 mM HEPES pH 7.4, 0.1 M NaCl, 0.5 mM TCEP completed purification. To purify full-length Periphilin, we followed the QIAexpressionist batch purification protocol under denaturing conditions (QIAGEN) followed by purification on a Superdex 200 column in 6 M urea, 0.2 M sodium phosphate pH 7.4, 20 mM TRIS pH 7.4, 1 mM TCEP.

### X-ray crystallography

Crystals were grown at 18°C by sitting drop vapor diffusion. Purified Periphilin-TASOR complex was mixed with an equal volume of reservoir solution: 0.1 M Citrate pH 4.5, 1 M ammonium sulfate. Crystals were harvested into a 70:30 mix of mother liquor to protein buffer supplemented with 20% DMSO with or without 1 M NaBr. Crystals were frozen in liquid nitrogen. X-ray diffraction data were collected at 100 K at Diamond Light Source (DLS) beamline I04-1. Automatic experimental phasing pipelines implemented at DLS including CRANK2 (28) determined phases with the single anomalous dispersion (SAD) method using bromine as the heavy atom. A polyalanine model built with CRANK2 was used as a molecular replacement search model for the native dataset (without NaBr) in PHENIX (29). The atomic model was built with COOT (30) and iteratively refined with PHENIX (29) at 2.5 Å resolution. See Table 1 for data collection and refinement statistics.

**Table 1.** Crystallographic data collection and refinement statistics for the minimal core Periphilin–TASOR complex

Data collection	Native	Br derivative
X-ray source	DLS I04-1	DLS I04-1
Space group	*P3_2_21*	*P3_2_21*
Cell dimensions		
*a* = *b*, *c* (Å)	93.57, 84.97	93.96, 82.30
α = β, γ (°)	90, 120	90, 120
Wavelength (Å)	0.91587	0.91587
Resolution (Å)^a^	41.0–2.51 (2.58–2.51)	81.4–2.93 (3.01–2.93)
Observations	150 312	183 145
Unique reflections	15 065	9350
*R*_merge_^b^	0.081 (1.014)	0.088 (2.426)
*R*_pim_^c^	0.029 (0.360)	0.020 (0.576)
<*I*> / σ *I*	15.0 (2.1)	25.5 (1.4)
Completeness (%)	99.9 (100)	100 (100)
Multiplicity	5.1 (4.9)	10.5 (9.6)
CC(1/2)	0.998 (0.735)	0.999 (0.542)
**SAD phasing with Br**		
CC_ano_		0.57 (0.02)
|D_ano_|/σD_ano_		1.56 (0.71)
Figure of merit, FOM (initial)		0.36
FOM (density modified)		0.43
FOM (after autobuild)		0.65
**Refinement**		
Resolution (Å)	41.0–2.51	
*R*_work_/*R*_free_^d^	0.228 / 0.271	
No. of non-H atoms		
Protein	1689	
Sulfate Ions	1	
Water	0	
No. riding H atoms	1707	
Mean *B*-factor (Å^2^)^e^	108	
MolProbity Clashscore	3.23	
RMS^f^ deviations		
Bond lengths (Å)	0.014	
Bond angles (°)	1.393	
Ramachandran plot		
% favored	96.53	
% allowed	2.48	
% outliers	0.99	
PDB code	PDB: 6SWG	
Diffraction data	DOI:15785/SBGRID/714	

^a^Highest resolution shell is shown in parentheses.

^b^ *R*_sym_ = Σ_*hkl*_Σ_i_ |*I_hkl,i_* – <*I*>_*hkl*_| / Σ_*hkl*_Σ_*i*_|*I_hkl,i_*|, where *I_hkl_* is the intensity of a reflection and _*hkl*_ is the average of all observations of the reflection.

^c^ *R*_pim_ = Σ_*hkl*_ (*N_hkl_* – 1)^−1/2^ × Σ_*i*_ |*I_hkl,i_* – <*I*>_*hkl*_| / Σ_*hkl*_Σ_*i*_|*I_hkl,i_*|, where *I_hkl_* is the intensity of a reflection and _*hkl*_ is the average of all observations of the reflection.

^d^ *R*_free_, *R*_work_ with 5% of *F*_obs_ sequestered before refinement.

^e^Residual *B*-factors after TLS refinement. See PDB entry for TLS refinement parameters.

^f^R.M.S., root mean square.

### Size-exclusion chromatography and multi-angle light scattering (SEC-MALS) analysis

100 μl of protein sample was subjected to SEC at 293 K using a Superdex 200 10/300 column (GE Healthcare) pre-equilibrated in PBS at a flow rate of 0.5 ml min^−1^. The SEC system was coupled to a multi-angle light scattering (MALS) module (DAWN-8+, Wyatt Technology). Molar masses of peaks in the elution profile were calculated from the light scattering and protein concentration, quantified using the differential refractive index of the peak assuming a dn/dc of 0.186, using ASTRA6 (Wyatt Technology).

### Immunofluorescence microscopy

Cells were grown on glass cover slips and then fixed with 4% formaldehyde in PBS for 15 min. Cells were permeabilized with 0.1% Triton X100 in PBS and then blocked with 5% BSA in PBS. Samples were stained with primary anti-Periphilin antibody (Atlas, HPA038902) at dilution 1:500 for 1 h and after washing with blocking buffer with secondary anti-rabbit AlexaFluor 568 antibody diluted 1/500 for 1 h. Cover slips were mounted on microscopy glasses with ProLong Gold anti-fade reagent with DAPI (Invitrogen). Imaging was performed using Nikon Ti microscope equipped with CSU-X1 spinning disc confocal head (Yokogawa) and with Zeiss 780 system.

### CUT&RUN H3K9me3 profiling

We followed the protocol detailed by Henikoff and colleagues (31). Briefly, 250 000 cells (per antibody/cell line combination) were washed twice (20 mM HEPES pH 7.5, 0.15 M NaCl, 0.5 mM spermidine, 1× Roche complete protease inhibitors) and attached to ConA-coated magnetic beads (Bangs Laboratories) pre-activated in binding buffer (20 mM HEPES pH 7.9, 10 mM KCl, 1 mM CaCl_2_, 1 mM MnCl_2_). Cells bound to the beads were resuspended in 50 μl buffer (20 mM HEPES pH 7.5, 0.15 M NaCl, 0.5 mM Spermidine, 1x Roche complete protease inhibitors, 0.02% w/v digitonin, 2 mM EDTA) containing primary antibody (1:100 dilution). Incubation proceeded at 4°C overnight with gentle shaking. Tubes were placed on a magnet stand to remove unbound antibody and washed three times with 1 ml digitonin buffer (20 mM HEPES pH 7.5, 0.15 M NaCl, 0.5 mM Spermidine, 1× Roche complete protease inhibitors, 0.02% digitonin). pA-MNase (35 ng per tube, a generous gift from Steve Henikoff) was added in 50 μl digitonin buffer and incubated with the bead-bound cells at 4°C for 1 h. Beads were washed twice, resuspended in 100 μl digitonin buffer and chilled to 0–2°C. Genome cleavage was stimulated by addition of 2 mM CaCl_2_ (final), briefly vortexed and incubated at 0°C for 30 min. The reaction was quenched by addition of 100 μl 2× stop buffer (0.35 M NaCl, 20 mM EDTA, 4 mM EGTA, 0.02% digitonin, 50 ng/μl glycogen, 50 ng/μl RNase A, 10 fg/μl yeast spike-in DNA (a generous gift from Steve Henikoff)) and vortexing. After 10 min incubation at 37°C to release genomic fragments, cells and beads were pelleted by centrifugation (16 000 g, 5 min, 4°C) and fragments from the supernatant purified with a Nucleospin PCR clean-up kit (Macherey-Nagel). Illumina sequencing libraries were prepared using the Hyperprep kit (KAPA) with unique dual-indexed adapters (KAPA), pooled and sequenced on a NovaSeq6000 instrument. Paired-end reads (2 × 150) were aligned to the human and yeast genomes (hg38 and R64-1-1 respectively) using Bowtie2 (–local –very-sensitive-local –no-mixed –no-discordant –phred33 -I 10 -X 700) and converted to bam files with samtools. Conversion to bedgraph format and normalization was performed with bedtools genomecov (-bg -scale), where the scale factor was the inverse of the number of reads mapping to the yeast spike-in genome. CUT&RUN experiments to assess H3K9me3 regulation by Periphilin variants were done in two independent replicate experiments. Peaks defined as HUSH-regulated were reported elsewhere (17). Normalized bigwig files were generated (UCSC), displayed in IGV (Broad Institute) and heatmaps plotted with computeMatrix and plotHeatmap commands (deepTools). Figures were prepared in Inkscape.

### Periphilin solubility assays

Denatured protein samples in denaturing buffer (6 M urea, 0.2 M sodium phosphate pH 7.4, 20 mM TRIS pH 7.4, 1 mM TCEP) were concentrated to 120 μM protein on a 10-kDa cutoff centrifugal concentrator. Solutions with increasing concentrations of urea were prepared by diluting 1 μl Periphilin into 30 μl buffer. The absorbance at 400 nm (OD_400_) was measured in duplicate on a ClarioSTAR plate reader (BMG Labtech).

### Differential interference contrast (DIC) microscopy

WT and Δ1-127 Periphilin variants in Denaturing Buffer (see above) were diluted into 0.5 M urea buffer (0.5 M urea, 0.2 M sodium phosphate pH 7.4, 20 mM TRIS pH 7.4, 1 mM TCEP). After 1 h incubation, 5 μl of each sample was applied to a glass dish (Ibidi) and imaged on a Nikon Ti2 microscope with a 100× objective.

### Statistics

No statistical methods were used to predetermine sample size, experiments were not randomized, and the investigators were not blinded to experimental outcomes. Reporter silencing assays were performed at least three times in independent experiments. Repression activity data are represented as the mean ± standard error of the mean (s.e.m.), calculated with PRISM 8 (GraphPad), with three biological replicates for all experiments except WT with ten replicates.

## RESULTS

### Both N- and C-terminal regions of Periphilin are required for HUSH function

**Figure 1.**
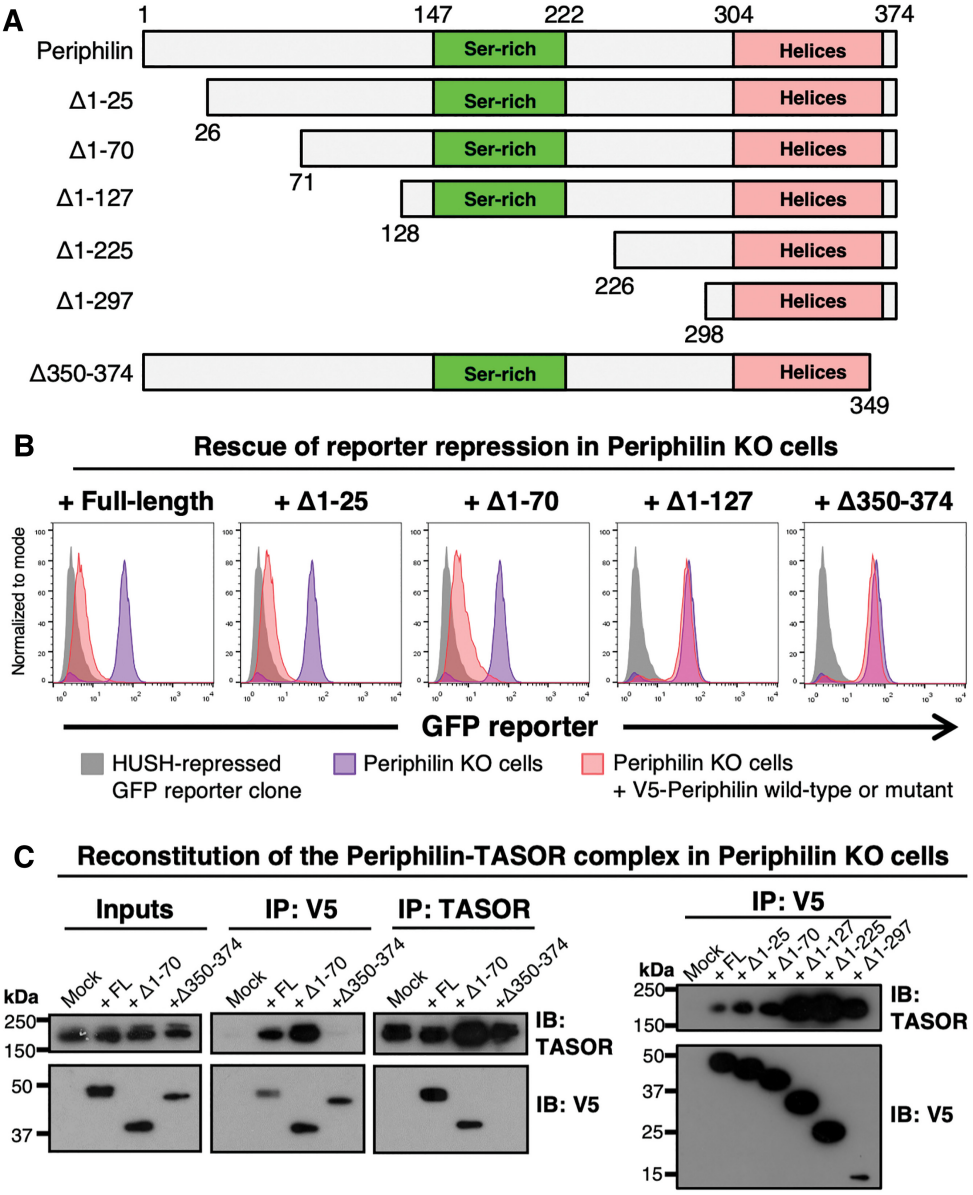
Both N- and C-terminal regions of Periphilin are required for HUSH function but only the C-terminal region is required for HUSH complex formation. (**A**) Schematic representation of Periphilin constructs used for complementation assay. All variants were expressed with an N-terminal V5 tag. (**B**) Repression of a lentiviral GFP reporter in Periphilin KO cells complemented with full-length Periphilin or truncation mutants (data shown 7 days post-transduction). The Δ1–127 and Δ350–374 variants fail to rescue reporter repression. (**C**) Pulldown assays with Periphilin and TASOR, the largest HUSH component. Periphilin and TASOR were immunoprecipitated (IP) from Periphilin KO cells complemented with Periphilin deletion mutants on Protein A/G resin decorated with anti-V5 or anti-TASOR primary antibody, respectively. TASOR and Periphilin proteins bound to the resin were quantified by Western immunoblot (IB). Only the C-terminal Periphilin deletion (Δ350–374) abolished TASOR binding. The V5 tag was used to detect Periphilin.

To identify the subdomains of Periphilin required for HUSH function, we generated various Periphilin constructs with N- or C-terminal truncations and assessed their silencing activity as part of the HUSH complex (Figure 1A). We used the 374-amino acid isoform 2 of Periphilin (UniProt Q8NEY8-2) as the reference sequence in this study rather than the longer isoform 1 (UniProt Q8NEY8-1), as isoform 2 fully restores HUSH function in Periphilin-deficient cells (11). Repression of a lentiviral GFP reporter in Periphilin knockout (Periphilin KO) HeLa cells was used as a measure of silencing activity. As reported previously (11), GFP reporter expression was repressed in wild-type cells and derepressed in Periphilin KO cells (Figure 1B). Transduction of Periphilin KO cells with a Periphilin construct lacking amino acids 1–25 (Δ1–25) or 1–70 (Δ1–70) rescued reporter expression to the same extent as transduction with wild-type Periphilin. However, transduction with Periphilin constructs lacking amino acids 350–374 (Δ350–374) or 1–127 (Δ1–127) failed to rescue reporter repression in Periphilin KO cells, indicating that both N- and C-terminal regions of Periphilin are required for HUSH function.

The loss of HUSH function with the Δ350–374 and Δ1–127 Periphilin mutants could be due to loss of an intrinsic activity of Periphilin or failure of Periphilin to be recruited to the HUSH complex. To distinguish between these, we measured coprecipitation of Periphilin and TASOR in pulldown assays. Periphilin and TASOR were purified on an immunoaffinity resin (immunoprecipitated) from lysates of Periphilin KO cells complemented with Periphilin deletion mutants. TASOR coeluted with all N-terminal deletion mutants tested, up to Δ1–297 (Figure 1C). Conversely, wild-type and Δ1-70 Periphilin both coeluted with TASOR. In contrast, TASOR did not associate with immunoprecipitated Δ350–374 Periphilin and Δ350–374 Periphilin did not associate with TASOR. Hence, only the C-terminal region of Periphilin is required for binding to TASOR, and the N-terminal region of Periphilin must have other properties necessary for HUSH function.

### Structure of the core Periphilin-TASOR complex identifies interfaces required for HUSH function

**Figure 2.**
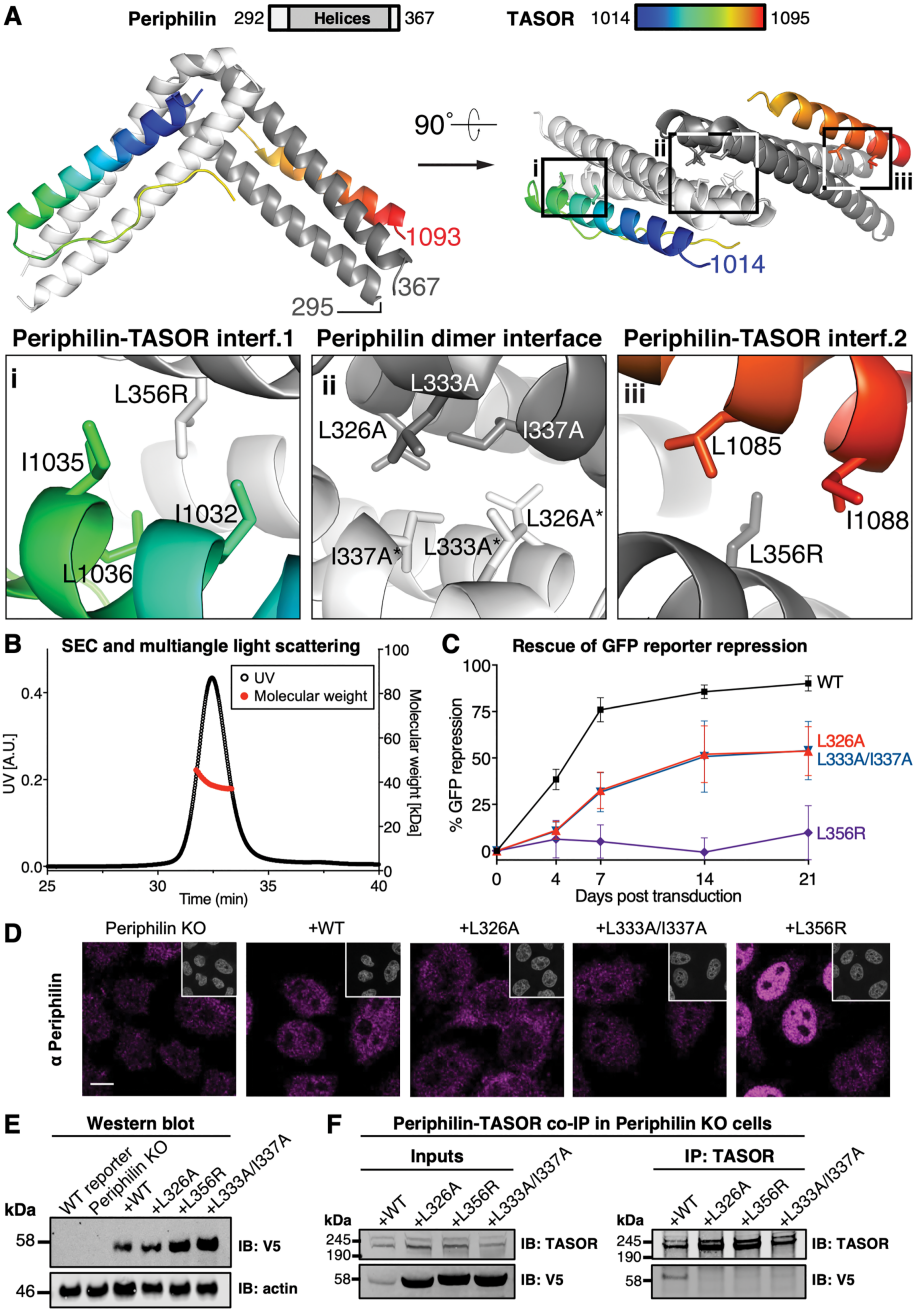
Periphilin and TASOR form a 2:1 complex required for HUSH function. (**A**) Crystal structure of the minimal Periphilin–TASOR core complex. The Periphilin fragment (residues 292–367, light/dark grey) forms a homodimer of helical hairpins. The TASOR fragment (residues 1014–1095, rainbow colors) wraps around the Periphilin dimer, adding an α-helix to each Periphilin hairpin to form two helical coiled coils. Insets show close-up views of the Periphilin-TASOR interfaces (‘i’, ‘iii’) and the Periphilin dimer interface (‘ii’). Residues forming key contacts and mutations designed to disrupt Periphilin-TASOR complex formation are labeled. (**B**) SEC-MALS of minimal Periphilin-TASOR core complex. The molecular weight calculated from light scattering data is consistent with a 2:1 complex in solution. (**C**) Repression of a lentiviral GFP reporter in Periphilin KO cells complemented with Periphilin mutants designed to inhibit Periphilin-TASOR complex assembly. Reporter expression was monitored over 21 days by flow cytometry. The log_10_(GFP fluorescence) data for live cells were converted to percent repression activity with WT HeLa reporter cells set at 100% and Periphilin KO cells set a 0% repression (see Materials and Methods). (**D**) Immunofluorescence microscopy of Periphilin KO cells transduced with Periphilin mutants affecting Periphilin–TASOR complex assembly. Cells were fixed 4 days post-transduction and stained with anti-Periphilin antibody (magenta) and DAPI (grey, insets). Scale bar, 10 μm. (**E**) Western immunoblot of Periphilin KO cells (whole cell lysate, 1% SDS) transduced with the three mutants shown in (**C**). The V5 tag was used for detection of Periphilin 7 days after transduction. Comparison with the actin loading control (lower panel) shows the mutants are expressed at higher levels than wild-type. (**F**) Pulldown assay with TASOR and the Periphilin mutants shown in (**C**). TASOR was immunoprecipitated (IP) from Periphilin KO cells (14 000 g cell lysate supernatant, 1% NP-40) complemented with V5-tagged Periphilin mutants on resin decorated with anti-TASOR antibody. TASOR and Periphilin proteins bound to the resin were quantified by Western immunoblot (IB), using the V5 tag to detect Periphilin. All three mutations abolished TASOR binding despite being more abundant than wild-type in the cell lysate supernatants.

Having established that the C-terminal region of Periphilin (residues 297–374) is required for binding to TASOR, we sought to identify the structural determinants of Periphilin-TASOR assembly. We recently mapped the Periphilin binding region in TASOR to a small region within residues 1000–1085 (17). Initial attempts to crystallize Periphilin–TASOR complexes failed until we determined that residues 285–291 and 368–374 of Periphilin were disordered, exploiting partial assignment of NMR spectra with ^15^N- and ^13^C-labeled Periphilin (Supplementary Figure S1). Thus, a crystal structure of a Periphilin fragment spanning residues 292–367 bound to a TASOR fragment spanning residues 1014–1095 was determined using the single anomalous dispersion (SAD) phasing method, with bromine as the anomalous scatterer (Table 1). The structure contains two Periphilin molecules and a single TASOR molecule (Figure 2A). The Periphilin fragments form helical hairpins with a mixture of α-helix and 3_10_-helix secondary structure. The two Periphilin hairpins pack against each other via a 118 Å^2^ hydrophobic interface formed by the hydrophobic side chains of Leu326, Leu333 and Ile337. The resulting Periphilin homodimer has twofold symmetry. The TASOR molecule forms two α-helices that wrap around the outer surfaces of the Periphilin dimer. The TASOR helices add a third helix to each Periphilin helical hairpin to form two three-helix coiled-coils. Each TASOR helix forms leucine zipper-type hydrophobic contacts, which typify helical coiled-coils. Unusually, however, each Periphilin subunit binds to a different TASOR sequence (residues 1014–1052 and 1072–1093, respectively) with an identical binding surface (Figure 2A). Notably residues 1055–1071 of TASOR, between the two Periphilin-binding segments, are disordered, but these 17 residues could easily span the 35–40 Å trajectory needed to connect residues 1054 and 1072 in the Periphilin-TASOR complex. Binding of TASOR to Periphilin buries a total of 428 Å^2^. The 2:1 stoichiometry of the Periphilin-TASOR core complex was confirmed in solution by size-exclusion chromatography coupled with multiangle light scattering (SEC-MALS; Figure 2B) and non-denaturing mass spectrometry (Supplementary Figure S2).

To determine whether the binding interfaces observed in the Periphilin-TASOR complex are required for HUSH function, we used our structure to design point mutations in Periphilin predicted to interfere with Periphilin-TASOR complex assembly and measured the silencing activity of the mutants in the GFP reporter assay described above. Variants L326A and L333A/I337A were generated to target the Periphilin dimer interface; Periphilin L356R was generated to target both Periphilin-TASOR interfaces (Figure 2A). The L356R variant failed to rescue reporter repression in Periphilin KO cells, whereas the L326A and L333A/I337A variants each had approximately half of the repression activity of wild-type Periphilin (Figure 2C). Immunofluorescence microscopy with an anti-Periphilin antibody confirmed that all variants were expressed with the same nuclear localization as wild-type Periphilin (Figure 2D). Immunoblots against the V5 tag on Periphilin showed that the variants were expressed at higher levels than wild-type Periphilin (Figure 2E).

To determine whether the engineered mutations inhibited Periphilin-TASOR complex formation, we measured coprecipitation of Periphilin and TASOR in a pulldown assay. TASOR was purified on an immunoaffinity resin from lysates of Periphilin KO cells complemented with the Periphilin mutants. All three variants failed to bind TASOR despite being present at higher levels than wild-type Periphilin in the input cell lysate supernatant (Figure 2F). The partial repression activity of L326A and L333A/I337A suggests that these variants may have residual TASOR binding affinity inside the cell, where conditions are more conducive to binding than in the pulldown assay. We conclude that the leucine zipper interactions at the Periphilin dimer and Periphilin–TASOR interfaces are required for HUSH function and that the minimal core Periphilin:TASOR complex has a 2:1 stoichiometry. Whether fully active HUSH complex with full-length subunits contains a Periphilin homodimer and a single TASOR molecule or forms higher-order assemblies in the nucleus remains to be determined.

### Sequences with predicted disorder in the Periphilin N-terminal region required for HUSH function

**Figure 3.**
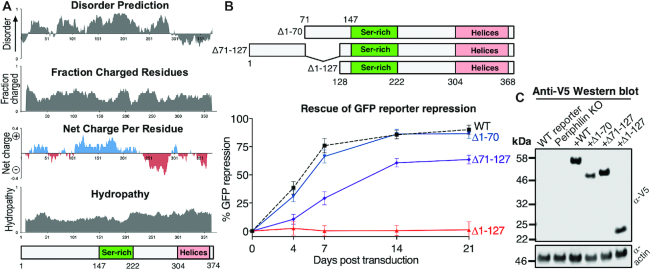
The NTD of Periphilin required for HUSH function contains partially-redundant sequences predicted to be unstructured. (**A**) Predicted structural disorder, charge and hydropathy of Periphilin calculated with localCIDER (27). (**B**) Repression of a lentiviral GFP reporter in Periphilin KO cells complemented with Periphilin variants containing deletions in the NTD. Repression activity is calculated as in Figure 2C. The WT curve (dotted line) is shared with contemporaneous experiments reported in Figure 2C. (**C**) Western blot of Periphilin KO cells transduced with the Periphilin N-terminal deletion variants, using the V5 tag for detection 7 days after transduction. The three mutants are expressed at similar levels.

The requirement of Periphilin residues 1–127 for HUSH-dependent silencing but not HUSH complex assembly (Figure 1) raises the question of how this N-terminal domain (NTD) contributes to HUSH function. Residues 20–291 of Periphilin are predicted to be unstructured (Figure 3A). The sequence is more polar than hydrophobic, with clusters of alternating positive and negative net charge but an approximately neutral overall net charge. The NTD of Periphilin has a greater than average number of serine, arginine, tyrosine and negatively-charged residues (see Figure 5A below). Residues 147–222 (140-215 in isoform 1) are a serine-rich domain with six candidate serine phosphorylation sites, and a further three candidate sites at nearby residues 117, 121 and 140 (32–34). To shed light on the role of these elements in HUSH-dependent silencing we generated Periphilin variants with various deletions in the NTD and measured their reporter repression activity over 21 days. Consistent with the reporter repression data shown in Figure 1A, the Δ1-70 mutant repressed reporter expression to the same extent and at the same rate as wild-type Periphilin, whereas the Δ1-127 variant had no repression activity (Figure 3B). Unexpectedly, however, addition of residues 1–70 to the Δ1-127 variant restored repression activity to 70% of wild-type activity. Hence, deletion of Periphilin residues 1–70 does not affect HUSH activity but these residues restore activity if residues 71–127 are deleted. Western blots confirmed that all variants were expressed at similar levels (Figure 3C). We conclude that the presence of either residues 1–70 or 71–127 is sufficient to confer significant HUSH-dependent silencing activity, but neither segment is essential. Together, the amino acid sequence and partially-redundant activities of the Periphilin NTD suggest that it is intrinsically disordered and hence that its contribution to HUSH activity stems from primary sequence attributes rather than tertiary structure.

### The NTD and TASOR-binding site are required for H3K9 methylation at HUSH-regulated loci

**Figure 4.**
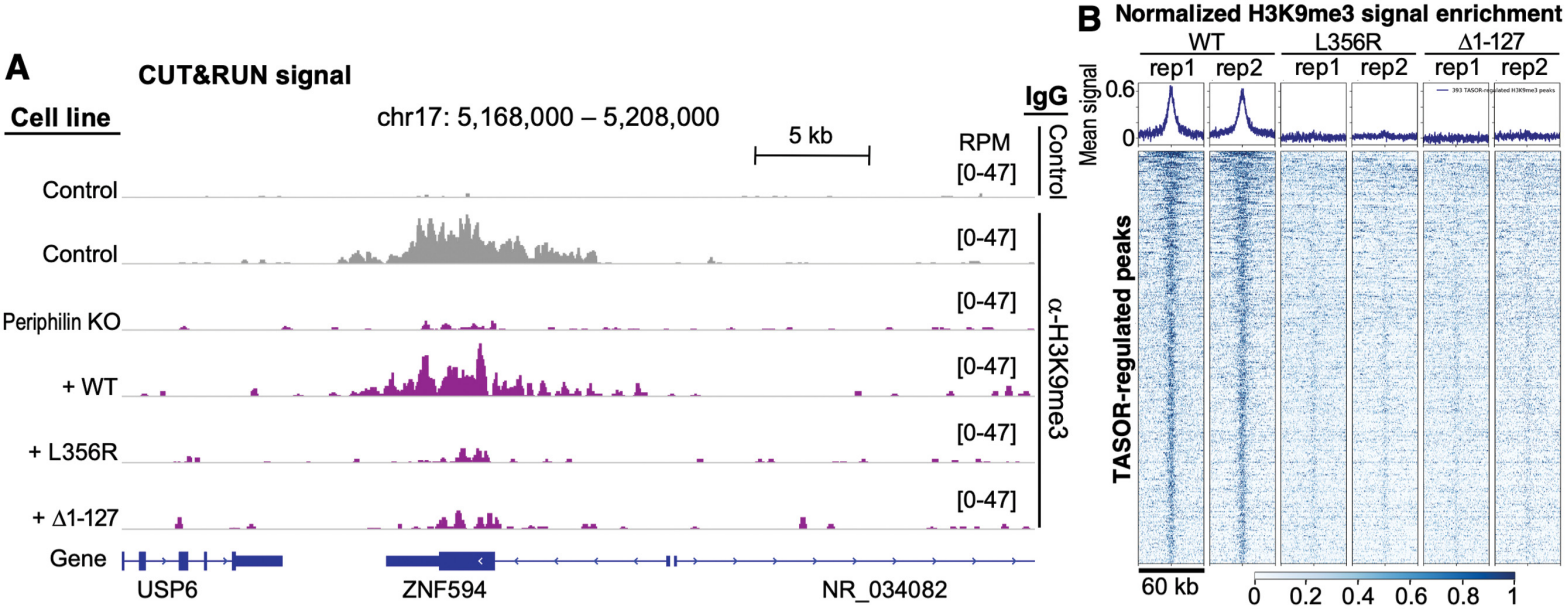
Genome-wide analysis of H3K9me3 distribution in cells expressing wild-type and functionally deficient variants of Periphilin. (**A**) Genome browser snapshot of H3K9me3 distribution in the presence of different Periphilin variants. H3K9me3 distribution is shown at the *ZNF594* locus, shown previously to be transcriptionally repressed by HUSH (11). Other representative snapshots are shown in Supplementary Figure S3. An H3K9me3 track from parent HeLa cells (Control) and a track with a non-cognate IgG are shown in grey as positive and negative controls, respectively (17). The Periphilin-complemented tracks are in purple. All experiments were run in duplicate with similar results. RPM, scaled reads per million. (**B**) Heatmap showing CUT&RUN signal enrichment (normalized signal from complementing Periphilin construct minus normalized signal from Periphilin KO) over the 393 TASOR-regulated H3K9me3 peaks in the genome (17), centered on each peak, with a ±30 kb window. Both replicates are shown for WT, L356R and Δ1–127 Periphilin variants. The mean binned signal is shown above each heatmap. H3K9me3 is lost specifically over HUSH-regulated peaks, but is unaffected in HUSH-independent peaks (see Supplementary Figure S3C).

Deposition of the repressive epigenetic mark H3K9me3 by SETDB1 is an essential component of HUSH-dependent silencing (11). To assess the importance of the Periphilin N- and C-terminal regions in H3K9 trimethylation, we measured the genome-wide distribution of H3K9me3 in cells expressing different Periphilin variants with the CUT&RUN (Cleavage Under Targets and Release Using Nuclease) epigenomic profiling method (31). We found that in Periphilin KO cells H3K9 methylation was lost or markedly reduced at hundreds of loci, representing ∼1–2% of global H3K9me3 loci (Figure 4 and Supplementary Figure S3). The sites of H3K9me3 loss recapitulate those seen in previous ChIP-seq (chromatin immunoprecipitation followed by sequencing) studies on cells in which TASOR, MPP8 or Periphilin were knocked out – that is, a subset of ‘host’ gene exons and young intronic LINE-1 retrotransposons (11,16,17). Complementation of Periphilin KO cells with full-length wild-type Periphilin robustly restored H3K9me3 levels. However, the L356R TASOR-binding point mutant or the Δ1-127 NTD deletion mutant failed to restore H3K9 methylation at HUSH-regulated loci (Figure 4). This effect was specific: H3K9me3 levels were unaffected at HUSH-independent loci (Supplementary Figure S3). Taken together, the data indicate that both the disordered NTD and the folded TASOR-binding domain of Periphilin are required for HUSH-dependent H3K9 methylation.

### Arginine and tyrosine residues in the NTD contribute to HUSH function

**Figure 5.**
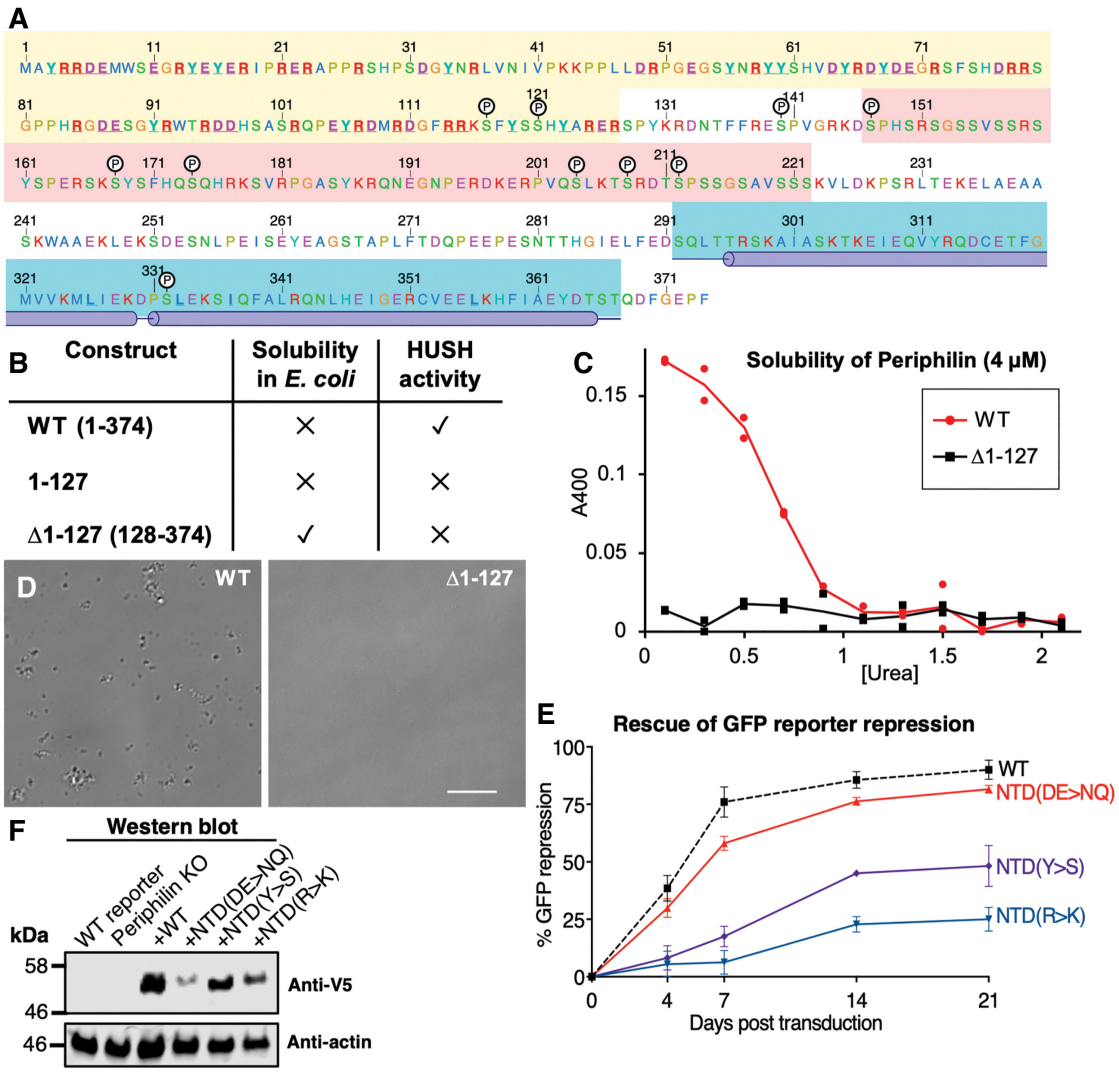
Physicochemical properties of the Periphilin NTD and contribution of enriched residues to HUSH activity. (**A**) Amino acid sequence of Periphilin-1 isoform 2 (UniProt Q8NEY8-2). Background shading color code: yellow, NTD; pink, Ser-rich region; blue, crystallized TASOR-binding region, with positions of the α-helices indicated. Candidate phosphorylation sites are labeled (P, black circles). Residues mutated in this study are underlined and in bold typeface. (**B**) Solubility of wild-type and terminal-deletion variants of Periphilin expressed in *E. coli*. The presence of HUSH-dependent silencing activity is indicated for each variant (see Figure 3B). (**C**) Solubility of WT and Δ1-127 Periphilin variants, measured as absorbance at 400 nm (A400) due to light scattering, as a function of urea concentration in the buffer. Representative of two independent experiments. (**D**) Differential interference contrast (DIC) microscopy of WT and Δ1-127 Periphilin variants (4 μM) in buffer containing 0.5 M urea. Scale bar, 10 μm. (**E**) Repression of a lentiviral GFP reporter in Periphilin KO cells complemented with Periphilin variants with all Asp/Glu, Arg or Tyr in the NTD mutated to Asn/Gln, NTD(DE>NQ); Lys NTD(R>K); or Ser NTD(Y>S), respectively. Repression activity is calculated as above. The WT curve (dotted line) is shared with contemporaneous experiments reported in Figure 2C. (**F**) Western blot against the V5 tag on Periphilin variants NTD(DE>NQ), NTD(Y>S) or NTD(R>K) 7 days after transduction into Periphilin KO cells. The NTD(DE>NQ) and NTD(R>K) variants are expressed at lower levels than wild-type.

**Figure 6.**
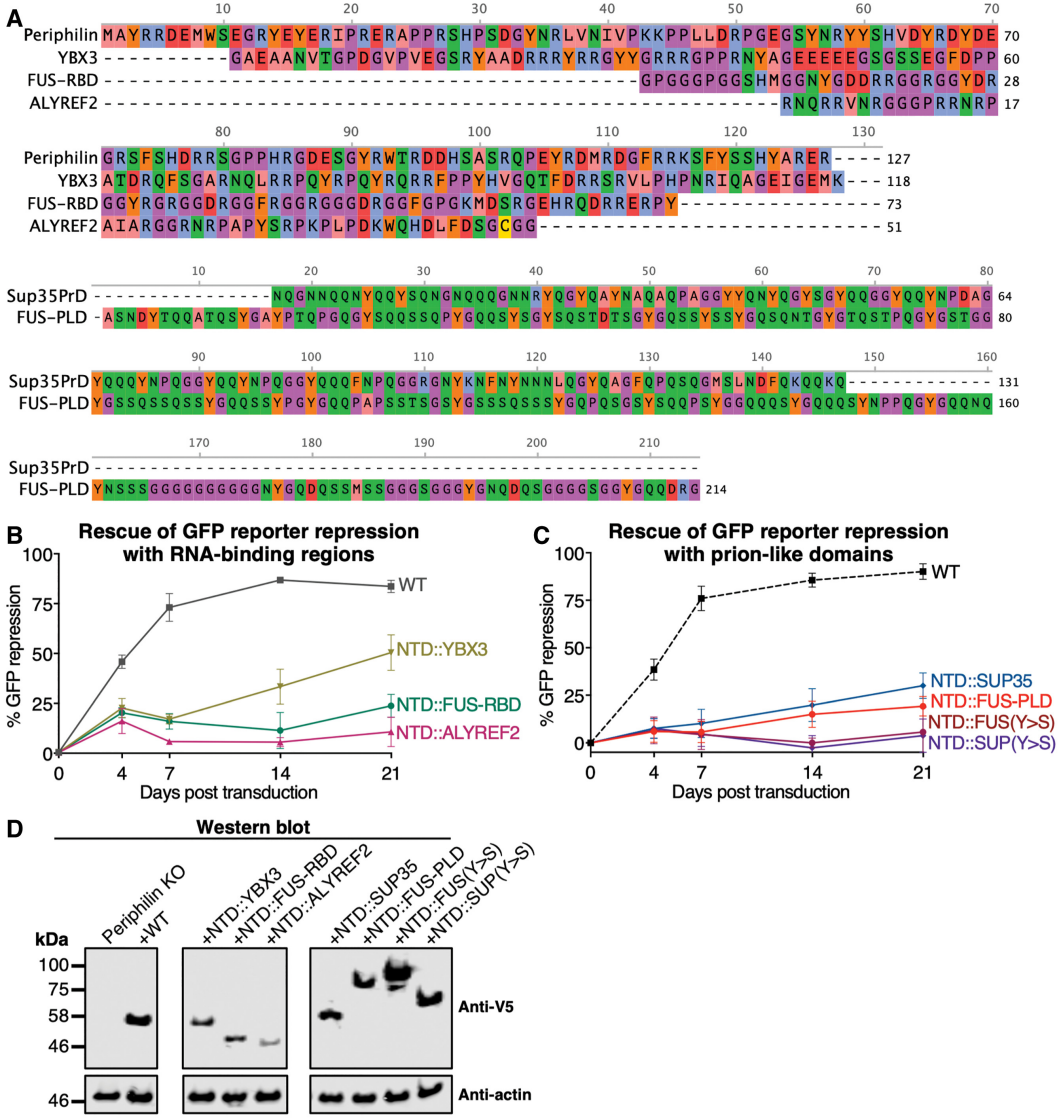
Complementation of NTD deletion with disordered regions from RNA-binding or prion-forming polypeptides partially rescues HUSH function. (**A**) Sequences used in this study to functionally complement the NTD of Periphilin in the Δ1-127 variant: YBX3, human Y-box-binding protein 3 residues 151–268, UniProt P16989-1; SUP35, *Saccharomyces cerevisiae* SUP35 prion domain, UniProt P05453 residues 5–135; FUS-RBD, human Fused in Sarcoma disordered RNA-binding region, UniProt P35637 residues 454–526; FUS-PLD, human Fused in Sarcoma prion-like low complexity domain, UniProt P35637 residues 2–214; ALYREF2, mouse Aly/RNA export factor 2 residues 17–67, UniProt Q9JJW6.1. (**B** and **C**) Repression of a lentiviral GFP reporter in Periphilin KO cells complemented with Periphilin variants with the NTD (residues 1–127) replaced by: (B) the disordered RNA-binding regions from ALYREF2, YBX3 or FUS; (C) the prion-like domain from FUS or the prion domain of SUP35, wild-type or with all tyrosine residues mutated to serine (Y>S). Residue ranges and sequence accession numbers are listed in (A). Repression activity is calculated as above. The WT curve in (C) is shared with contemporaneous experiments reported in Figure 2C and is shown here as a dotted line. The WT curve in (B) is part of a separate experiment and is represented as a solid line. (**D**) Western blot of Periphilin KO cells transduced with the Periphilin variants shown in (B) and (C). The variants were detected using their V5 tag 7 days post-transduction.

The physicochemical properties of the Periphilin NTD are reminiscent of the properties that govern the self-assembly of proteins into biomolecular condensates, in particular the Fused in Sarcoma (FUS) family of RNA-binding scaffold proteins. FUS family proteins contain N- and C-terminal intrinsically disordered regions with low sequence complexity resulting from a preponderance of specific subsets of amino acids (35). The N-terminal disordered region, known as the prion-like domain for its genetic association with prion-like inheritance in yeast and age-related neurodegenerative diseases in humans, is enriched in serine, glycine, tyrosine, glutamine, asparagine and proline (35,36). The C-terminal region comprises one or more folded RNA recognition motifs (RRMs) interspersed with low-complexity sequences enriched in arginine and glycine. Arginine-tyrosine interactions and π-stacking of tyrosine-containing strands into kinked β-sheet fibrils in these disordered regions can non-covalently crosslink the polypeptide chains into liquid- or gel-like condensates, which manifest in the cell as phase separations or membraneless compartments (37–40). The arginine-tyrosine interactions that promote phase separation of FUS family proteins are stabilized by complementary negative electrostatic charges from aspartate and glutamate residues in the prion-like domain (38). An excess of negative charge in FUS from multiple serine phosphorylation (or phosphomimetic mutations) decreases phase separation (41). The NTD of Periphilin contains a similar sequence bias as the disordered C-terminal regions of FUS family proteins, with a marked enrichment of serine, arginine, tyrosine, aspartate and glutamate residues (Figures 5A and 6A). Moreover, Periphilin has approximately the same number of positively and negatively-charged residues, and has 10 potential serine phosphorylation sites, a similar number as FUS.

To assess the potential of Periphilin to form condensates we expressed various Periphilin recombinant protein constructs in *E. coli*. Full-length Periphilin and a construct spanning the NTD alone (residues 1–127) were both insoluble and could not be purified from cell lysates under native conditions (Figure 5B). The Δ1–127 variant lacking the NTD was soluble but lacked repression activity as noted above. In contrast to FUS family proteins, which undergo phase separation and form hydrogels reversibly at low salt concentrations, Periphilin constructs containing the NTD remained insoluble even at higher than physiological salt concentrations (0.3 M NaCl). Full-length Periphilin could be solubilized and purified in the presence of 8 M urea but upon dilution of the urea to below 1 M the protein came out of solution, reversibly, forming solid aggregates detectable by absorbance in the visible light spectrum and by differential interference contrast microscopy (Figure 5C, D). Hence, the NTD, which is required for HUSH activity, induces Periphilin to self-aggregate without undergoing phase separation or hydrogel formation as seen in FUS family proteins.

Among the amino acids enriched in the disordered regions of Periphilin and FUS family proteins, tyrosine and arginine residues govern the phase separation properties of FUS family proteins (38). Mutation of tyrosine residues to serine, or arginine to alanine, in FUS disordered regions diminishes or abrogates phase separation and hydrogel formation (38,39). To determine whether arginine and tyrosine residues contribute to Periphilin self-aggregation, we generated Periphilin variants with all 24 arginine residues in the NTD mutated to lysine, NTD(R>K), or with all 13 tyrosine residues mutated to serine, NTD(Y>S) and measured the silencing activity of the mutants in our GFP reporter assay. Both variants reduced HUSH-dependent repression (Figure 5E). The NTD(R>K) variant, despite retaining the net charge of wild-type Periphilin, had <10% of wild-type activity 7 days post-transduction, and approximately one quarter of wild-type activity after 21 days. The NTD(Y>S) variant appeared less impaired, with 20% of wild-type activity after 7 days and 50% after 21 days. However, the NTD(Y>S) variant was expressed at significantly higher levels than the NTD(R>K) variant, suggesting that the impairment of the NTD(R>K) and NTD(Y>S) variants would be comparable at identical expression levels (Figure 5F).

Arginine-tyrosine interactions have been proposed to be stabilized by negatively-charged residues in FUS family proteins. Mutation of negatively-charged residues in FUS family proteins decreases their overall phase separation potential (38). In contrast, the negatively charged residues in the NTD were not required for silencing. Indeed, Periphilin variant NTD(DE>NQ) with all 22 aspartate or glutamate residues mutated to asparagine or glutamine, respectively, had repression activity similar to wild-type (Figure 5E) despite having a lower expression level than the wild-type, Y>S and R>K variants (Figure 5F).

### Disordered polypeptides with self-associating or RNA-binding properties partially complement NTD deletion

The similarity of the amino acid sequence bias in the Periphilin NTD and the disordered regions of RNA-binding domains from FUS family proteins raises the question of whether these low-complexity sequences have similar biophysical properties, which in Periphilin contribute directly to HUSH-dependent silencing. Insertion of the disordered portion of the RNA-binding domain from FUS (Figure 6A) into the Δ1–127 variant restored HUSH repression activity to approximately 25% of wild-type (Figure 6B, NTD::FUS-RBD). To determine whether self-association of a disordered polypeptide *per se* is sufficient to support HUSH silencing we generated variants containing the prion domain from yeast SUP35, or the prion-like domain of FUS in place of the NTD (NTD::SUP35 and NTD::FUS-PLD, respectively). Prion domains have a different sequence bias: enrichment of glutamine, asparagine and tyrosine and depletion of charged residues (Figure 6A) (36). Prion domains form highly stable steric zipper-type amyloid fibers distinct from the reversible associative polymers formed by FUS (37,42). Nevertheless, the NTD::SUP35 and NTD::FUS-PLD variants restored repression activity to 30% and 20% of wild-type (Figure 6C), respectively, suggesting prion-like aggregation partially functionally complements the NTD, to a comparable extent as the arginine/glycine-rich region of FUS.

Tyrosine residues are essential for the aggregation of FUS and the amyloidogenic properties of prion proteins (38,39,42,43). Mutation of all tyrosine residues to serine in the complementing sequences of FUS-PLD and SUP35 abrogated their silencing activity (Figure 6C, NTD::FUS(Y>S) and NTD::SUP(Y>S)).

A large proportion of proteins with low-complexity disordered regions bind RNA via arginine-rich sequences. Many of these RNA-binding proteins self-associate into liquid or gel phases, or form amyloid (or amyloid-like) fibers (44,45). The same arginine/glycine-rich regions of FUS family proteins that mediate phase separation also bind RNA, and RNA binding nucleates higher-order assembly of FUS (40). Moreover, prion proteins are strongly enriched for RNA-binding proteins (36). Formation of ribonucleoprotein complexes (RNPs)—in particular with mRNA—through phase separation or fibril formation is emerging as a central mechanism of co- and post-transcriptional regulation (44). To assess the potential contribution of RNA binding by Periphilin to silencing, we replaced the NTD with RNA-binding polypeptides from two RNA-binding proteins (Figure 6A), Y-box-binding protein 3 (YBX3) and Aly/RNA export factor 2 (ALYREF2). YBX3 a member of the cold shock domain (CSD) protein family that binds mRNA without sequence specificity via a disordered C-terminal tail rich in aromatic, basic and phosphorylated residues (46,47). YBX3 was recently shown to repress translation of certain mRNAs (48). ALYREF2 contributes to mRNA export by packaging mRNA into RNPs through interactions with an arginine-rich disordered N-terminal tail. The NTD::YBX3 variant restored HUSH repression activity to the greatest extent of any of the complementing sequences tested, with 50% of wild-type Periphilin 21 days post-transduction (Figure 6B). In contrast, the NTD::ALYREF2 variant did not restore repression. We note that the complementing YBX3 sequence and the Periphilin NTD both have a greater number of alternating positively and negatively-charged residues than the other complementing sequences (Figure 6A). Western blots showed that some of the complementing NTD sequences altered the expression levels: the inactive FUS(Y>S) sequence boosted expression and the ALYREF2, FUS-RBD and YBX3 sequences reduced expression relative to wild-type (Figure 6D). Efforts to measure RNA binding by Periphilin were hampered by the insolubility of purified protein constructs containing the NTD.

## DISCUSSION

We have identified the key structural and biochemical properties of Periphilin necessary for epigenetic silencing by the HUSH complex. The C-terminal coiled-coil domain directs HUSH complex assembly by dimerizing and binding TASOR through α-helical coiled-coil interactions. How the N-terminal region (NTD) of Periphilin contributes to silencing is more difficult to pinpoint due to its intrinsic structural disorder and its propensity to aggregate. We note that self-aggregation of the NTD correlates with HUSH function, as truncations that inhibit aggregation also inhibit silencing. As in self-associating disordered regions from many other proteins including FUS-family proteins, the NTD of Periphilin is enriched in tyrosine and arginine residues. These residues are required for HUSH transgene repression activity. Arginine-tyrosine π-stacking interactions are essential for the aggregation of FUS, and tyrosine residues contribute to the amyloidogenic properties of prion proteins. Hence, NTD self-association through arginine-tyrosine π-stacking interactions could play a role in HUSH silencing. Consistent with this notion, lysine failed to functionally substitute for arginine in silencing assays with our NTD(R>K) Periphilin variant.

Alongside the broad similarities between the Periphilin NTD and disordered regions from other arginine/tyrosine-rich proteins, the NTD has certain distinguishing properties. The sequence complexity of the NTD is not as low as in the disordered regions that drive phase separation of FUS family proteins. Periphilin lacks tyrosine residues flanked on both sides by serine or glycine to form [G/S][Y/F][G/S] motifs, which are hallmarks of fiber-forming Low-complexity Aromatic-Rich Kinked Segments (LARKS). Periphilin also contains a greater proportion of charged residues than FUS-family and prion proteins and may acquire further negative charges through phosphorylation. Moreover, the NTD induces the formation of solid aggregates rather than liquid-like phases, hydrogels or fibers. These distinguishing features may explain why the NTD was not fully complemented by the disordered regions from FUS or Sup35 in our HUSH silencing assays.

On balance, the similarities between the NTD and self-associating disordered regions from FUS-family proteins outweigh the differences. Indeed, counterbalancing the differences listed above, the NTD does contain sequences that resemble LARKS or are predicted to have amyloid-forming potential, for example SFYSSHYA, with a stacking free energy of –27 kcal/mol predicted by ZipperDB (49). Second, the negatively-charged residues in the NTD, though more abundant than in FUS and Sup35, are not essential for HUSH function. Furthermore, membraneless compartments formed by biomolecular condensates have been reported previously to have the characteristics of a solid rather than a liquid or gel (50,51). Hence, the most plausible mechanism for Periphilin NTD aggregation is via arginine-tyrosine π-stacking interactions, like FUS-family proteins but with greater cooperativity, resulting in a more abrupt transition from the soluble state to a solid aggregated state. Whether and how self-association via this mechanism translates into silencing activity in the HUSH complex remains unclear.

The biochemical properties of the NTD suggest that one of its functions in HUSH-dependent silencing may be to bind RNA. A majority of proteins with arginine-rich disordered regions bind RNA and self-associate into ribonucleoprotein (RNP) fibers or condensates (44,45). Polymerization of proteins— such as proteins from cold shock domain (CSD) family—on mRNA is emerging as a central mechanism to repress protein expression co- or post-transcriptionally (44). Notably, the disordered RNA-binding region of CSD-family protein YBX3, which binds certain mRNAs and represses their translation (48), functionally complemented the Periphilin NTD to a greater extent than any of the other sequences we tested. As in other CSD proteins, the disordered RNA-binding region of YBX3 is enriched in aromatic, basic and phosphorylated residues and binds mRNA without sequence specificity (46,47). Whether Periphilin forms RNPs with mRNA remains unknown. Binding of Periphilin to mRNA could explain the known propensity of HUSH to silence genes that are actively being transcribed (16,17). In support of HUSH binding to nascent transcripts, Periphilin and TASOR (as C3orf63) were identified in a proteomic screen for protein–mRNA interactions in human cells (26,52). Moreover, artificially increasing transcription of a transgene increases recruitment of HUSH to that locus (16). Why the HUSH complex preferentially binds to intronic LINE-1 elements within actively transcribed genes with some degree of sequence specificity (16,17), remains to be determined. We note that the cold shock domains of YBX3 and other Y-box binding proteins bind to specific sequences or structures in the untranslated regions of their target mRNAs (48,53). Although the HUSH complex does not appear to contain any classical RNA recognition motifs or cold shock domains (17), the possibility that TASOR or MPP8 contain a motif conferring specificity for RNA sequence or structure cannot be excluded.

The work presented and discussed here prompts us to propose the following model for how Periphilin may function in silencing. The NTD of Periphilin may bind nascent transcripts, with multiple Periphilin molecules binding to each target mRNA. The self-aggregation properties of NTD could then lead to the formation of large mRNPs. Transcripts within these mRNPs would be less accessible to transcription and translation machinery, thereby repressing expression. Other HUSH components or effectors, tethered via the C-terminal domain of Periphilin, could then sense and modify the epigenetic landscape or chromatin structure at the target site. This would include recruitment of chromatin-remodeling ATPase MORC2 and deposition of the transcriptionally repressive H3K9me3 mark by SETDB1. Further studies will be necessary to test and refine this model. This work provides a foundation to design new epigenetic therapies targeting HUSH to treat autoimmune diseases, cancer and retroviral infections.

## DATA AVAILABILITY

The structure factors and atomic coordinates were deposited in the Protein Data Bank with code PDB: 6SWG, DOI:10.2210/pdb6SWG/pdb. The original experimental X-ray diffraction images were deposited in the SBGrid Data Bank (data.SBGrid.org), with Data ID 714, DOI:10.15785/SBGRID/714. The CUT&RUN data were deposited in the Gene Expression Omnibus (GEO) database under accession code GSE155824, with additional controls and peak list information available in entry GSE155693.

## Supplementary Material

gkaa785_Supplemental_Files

## Notes

Present address: Daniil M. Prigozhin, Molecular Biophysics and Integrated Bioimaging Division, Lawrence Berkeley National Laboratory, Berkeley, CA 94720, USA.

Present address: Christopher H. Douse, Department of Experimental Medical Science, Lund University, Sölvegatan 19, Lund, Sweden.

Present address: Anna Albecka, MRC Laboratory of Molecular Biology, Francis Crick Avenue, Cambridge, UK.

Present address: Iva A. Tchasovnikarova, The Gurdon Institute, Tennis Court Road, Cambridge, UK.
