# Supplementary material for: Periphilin self-association underpins epigenetic silencing by the HUSH complex
Source: Nucleic Acids Res. 2020 Sep 25;48(18):10313–28. doi: 10.1093/nar/gkaa785 (PMC7544229; doi:10.1093/nar/gkaa785)
Supplement: gkaa785_Supplemental_Files [file gkaa785_supplemental_files.zip › Periphilin_v46_NAR_SupplData.pdf]

SUPPLEMENTARY DATA

SUPPLEMENTARY METHODS

Nuclear magnetic resonance (NMR) spectroscopy

A $^{1}H,^{15}N$ BEST-TROSY NMR spectrum of the Periphilin-TASOR complex ($^{15}N,^{13}C$-labeled Periphilin residues 285-374 with an N-terminal His_6 tag and TASOR residues 1014-1095) was acquired on a Bruker Avance 600 MHz spectrometer equipped with a triple resonance TCI cryoprobe at 298 K. Standard triple resonance experiments HNCA, CBCA(CO)NH, HNCACB, HNCO and HN(CA)CO enabled a partial resonance assignment of N- and C-terminal unstructured residues 285-291 and 368-374 of Periphilin. The largely reduced or absent signals for residues 292-367 is indicative for the increase in transverse relaxation of this structured region due complex formation with TASOR.

Liquid chromatography coupled to mass spectrometry (LC-MS)

Denatured Periphilin-TASOR (10 µM) was subjected to LC-MS analysis. Briefly, the complex was separated on a C4 BEH 1.7µm, 1.0 x 100 mm UPLC column (Waters, UK) using a modified NanoAcquity liquid chromatography unit (Waters, UK) to deliver a flow of approximately 50 µl $min^{-1.}$ The column was developed over 20 min with a 2-80% (v/v) gradient of acetonitrile in 0.1% (v/v) formic acid. The analytical column outlet was directly interfaced via an electrospray ionisation source, with a hybrid quadrupole time-of-flight mass spectrometer (Xevo G2, Waters, UK). Data were acquired over a m/z range of 300–2000, in positive ion mode with a cone voltage of 30 V. Scans were summed manually and deconvoluted with MaxEnt1 (Masslynx, Waters, UK).

Native mass spectrometry

10 µM Periphilin-TASOR complex was buffer-exchanged into 0.1 M ammonium acetate using P6 Bio-Spin columns (BioRad). The complex was analyzed on a SYNAPT G2Si HDMS mass spectrometer (Waters, UK). Briefly, 5 µl of protein was loaded into a GlassTip emitter (New Objective, USA) and sprayed into the instrument by nano-electrospray ionization (nano-ESI) with a voltage of 1.2 kV, cone voltage 150 V, offset 150 V and trap collision energy 40 V. Scans were summed and manually deconvoluted with MassLynx4.1 (Waters, UK).

SUPPLEMENTARY FIGURES

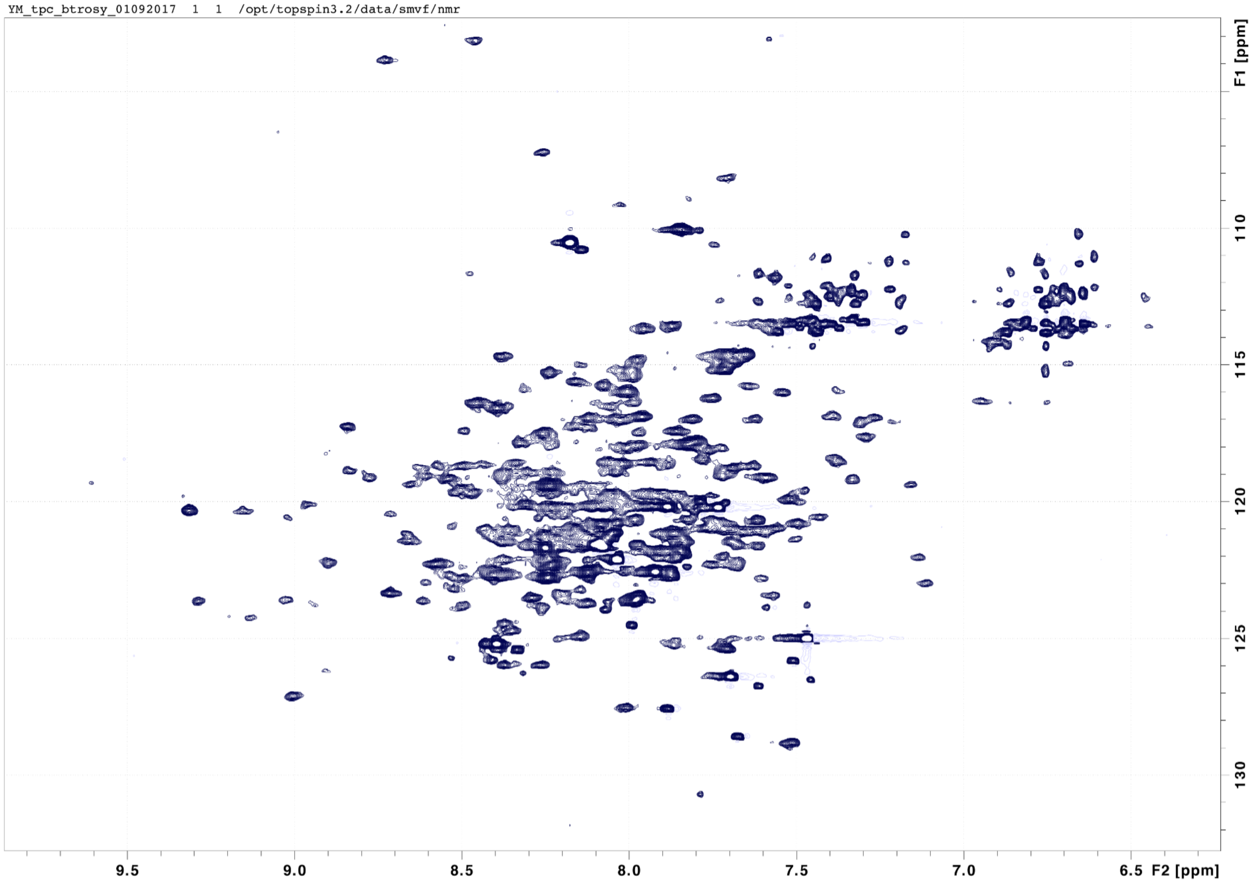

Supplementary Figure S1. $^{1}H,^{15}N$ BEST-TROSY nuclear magnetic resonance (NMR) spectrum of $^{15}N,^{13}C$-labeled Periphilin in the Periphilin-TASOR complex. The spectrum displays backbone $^{1}H,^{15}N$ correlations for labeled Periphilin. Large intensity variations are caused by the distinctly different dynamic behavior of unstructured residues. A partial resonance assignment of intense signals in the center of the spectrum corresponding to disordered residues 285-291 and 368-374 suggested that residues 292-367 of Periphilin were ordered.

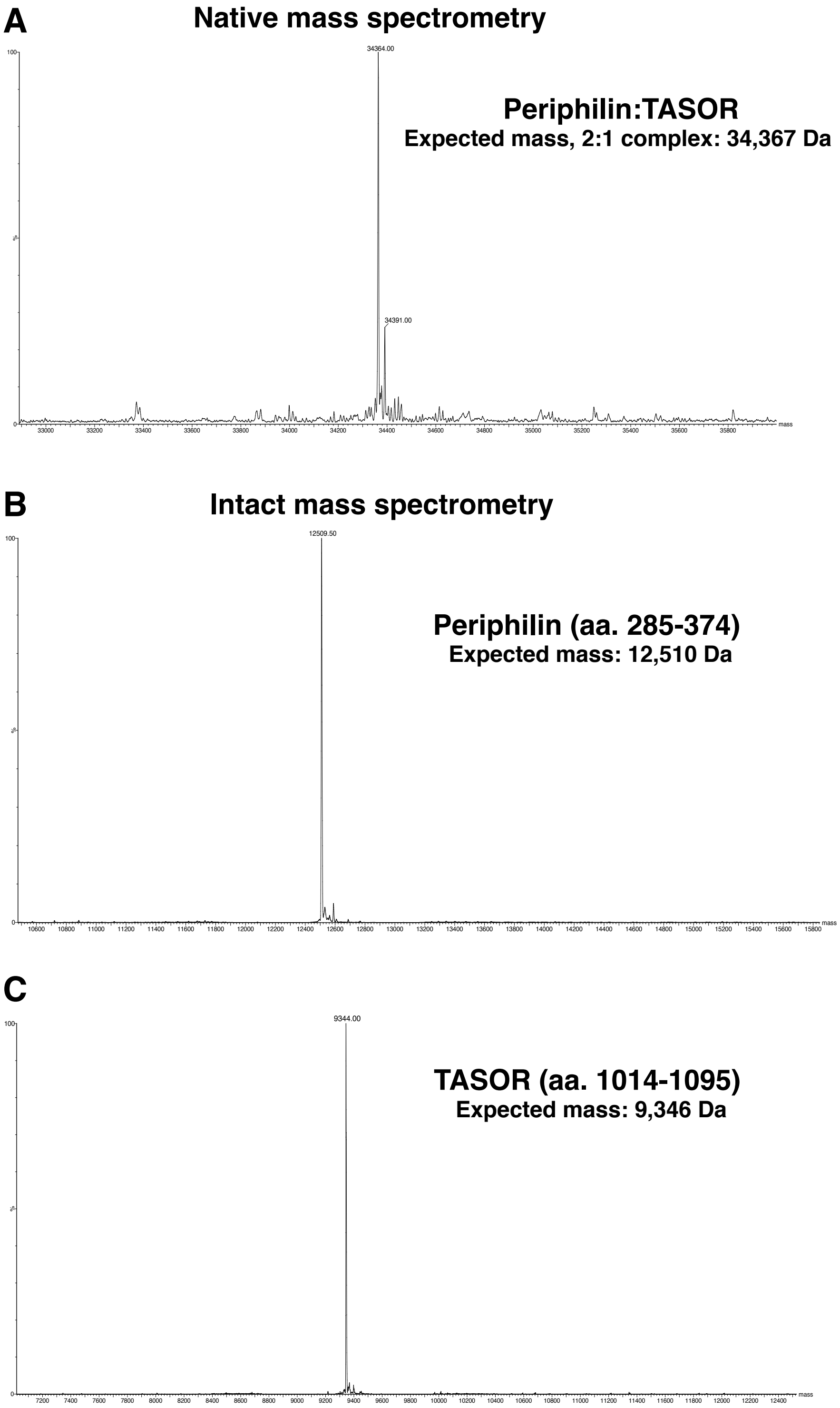

Supplementary Figure S2. Native and intact mass spectrometry of the Periphilin-TASOR complex and its components. (**A**) Deconvoluted native mass spectrum of the Periphilin-TASOR complex collected under non-denaturing conditions. (**B**) Deconvoluted intact mass spectrum of the TASOR-binding domain of Periphilin, residues 285-374 (denaturing conditions). (**C**) Deconvoluted intact mass spectrum of the Periphilin-binding domain of TASOR, residues 1014-1095 (denaturing conditions).

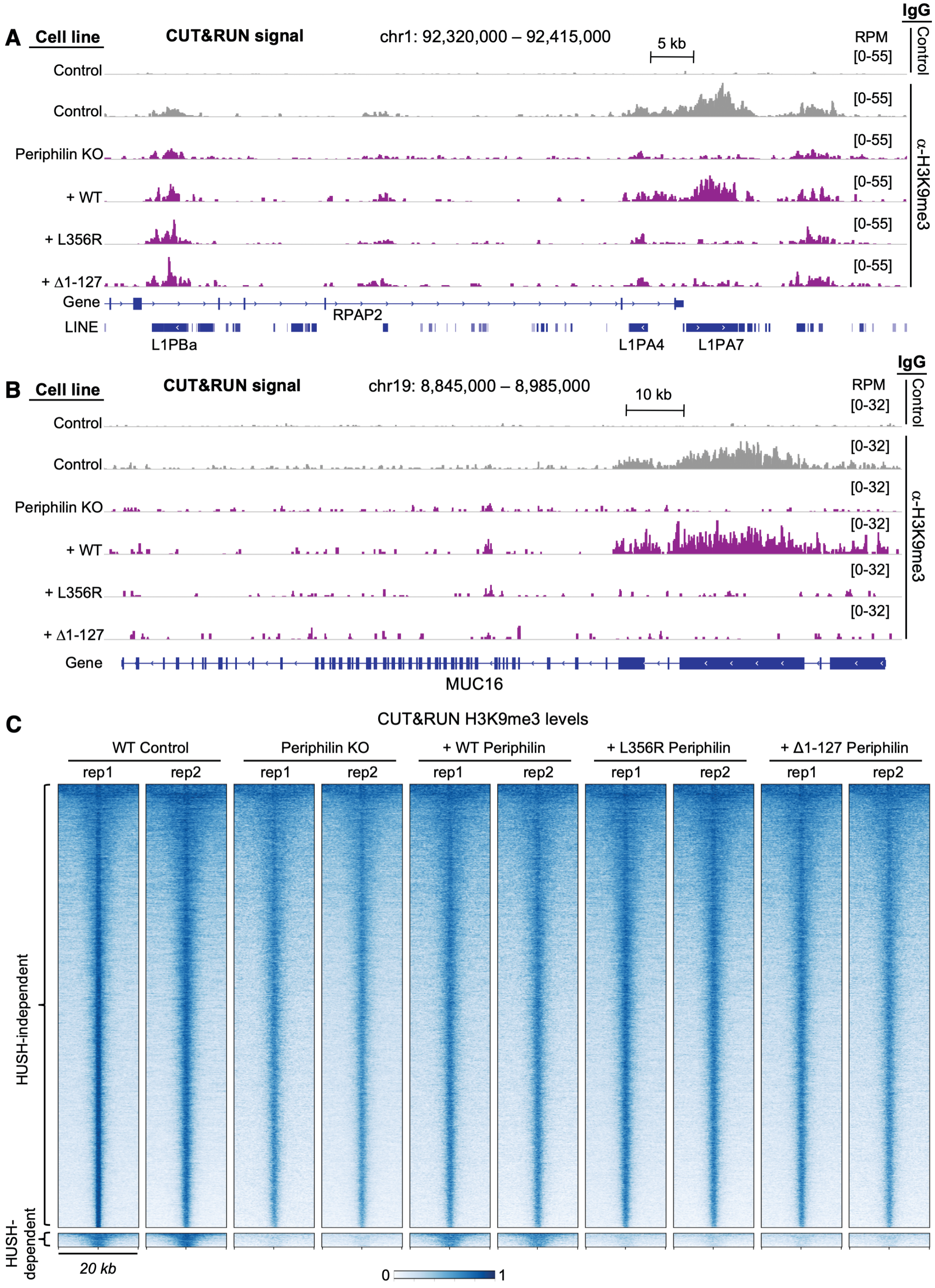

Supplementary Figure S3. CUT&RUN genome-wide analysis of Periphilin and H3K9me3 distribution with wild-type and functionally deficient variants of Periphilin. (**A**) and (**B**) Representative snapshots of H3K9me3

distribution along the genome in the presence of different Periphilin variants. H3K9me3 distribution is shown at a full-length (6kb) L1PA7 element, (**A**), and around gene MUC16 (**B**). Both loci were shown previously to be covered in HUSH-dependent H3K9me3. H3K9me3 from control cells and a track with a non-cognate IgG are shown in grey as positive and negative controls, respectively. The Periphilin-complemented tracks are in purple. Experiments were run in biological duplicate with similar results. RPM, reads per million, scaled to the total number of reads. (**C**) Heatmaps showing normalized CUT&RUN H3K9me3 signal from two replicate experiments in the indicated cell lines, globally over 12,573 HUSH-independent H3K9me3 peaks (top), and 393 HUSH-dependent peaks (bottom), centered on each peak, with a ±10 kb window. The loss of H3K9me3 signal in the Periphilin-deficient cell lines is specific for the HUSH-dependent loci. HUSH-dependent H3K9me3 peaks were defined by differential analysis of H3K9me3 levels in control and TASOR KO cells, as described (17).
